
JOURNAL INFORMATION
==============================
NLM Title Abbreviation: ScientificWorldJournal
Journal ID: ScientificWorldJournal
NLM Journal ID: 101131163
Journal ID: TSWJ

The Scientific World Journal

ISSN: 2356-6140
EISSN: 1537-744X
Publisher: Wiley

ARTICLE INFORMATION
==============================
PMCID: PMC5956385
PMID: 15349524
DOI: 10.1100/tsw.2004.42
Article ID: 813590
Article version: 1
Subjects: Research Article

Renal Trauma: A Practical Guide to Evaluation and Management

Brandes Steven B.
McAninch Jack W.
Department of Urology, University of California, San Francisco, CA, USA
Academic Editor: Anthony Atala

Publication date: 2004
Electronic publication date: 2004 Jun 7
Volume: 4
First page: 31
Last page: 40
Copyright: Copyright © 2004 Steven B. Brandes and Jack W. McAninch.
Copyright year: 2004
License: This is an open access article distributed under the Creative Commons Attribution License, which permits unrestricted use, distribution, and reproduction in any medium, provided the original work is properly cited.
License URL: https://creativecommons.org/licenses/by/3.0/

==============================
Article
*TheScientificWorldJOURNAL* (2004) 4 (S1), 31–40
ISSN 1537-744X; DOI 10.1100/tsw.2004.

The Scientific World
www.thescientificworld.com

### Renal Trauma: A Practical Guide to Evaluation and Management

Steven B. Brandes, MD and Jack W. McAninch, MD
*The Department of Urology, University of California San Francisco School of Medicine, San Francisco General Hospital, San Francisco, CA*

**E-mail**: brandess@msnotes.wustl.edu

*Republished from the Digital Urology Journal*

---

**DOMAIN**: urology

---

### INCIDENCE

At major urban trauma centers, the kidneys are injured in <5 % of all trauma cases. Renal trauma comprises about half of all genitourinary trauma. Since at most trauma centers blunt trauma is more common, blunt renal injuries thus occur as often as nine times as often as penetrating injuries.[1] Both kidneys are at equal disposition for injury.

### INDICATIONS FOR IMAGING

1. *Blunt trauma and gross hematuria.* Gross hematuria is the most reliable indicator for serious urological injury.[2] The degree of hematuria, however, does not correlate with the degree of injury. In fact, renal pedicle avulsion or acute thrombosis of segmental renal arteries can occur in the absence of hematuria while renal contusions can present with gross hematuria.[1]
2. *Blunt trauma, microscopic hematuria and shock.* Significant microscopic hematuria is >5 RBC/HPF in the first voided or catheterized specimen. Shock is any presence of systolic blood pressure <90 during transport or upon arrival in the emergency room. Blunt trauma patients with microhematuria and no shock have minor renal injuries in nearly all cases. Miller and McAninch,[1] based on findings in over 2000 blunt renal trauma injuries determined that in <0.2% of cases will a grade 2 or more severe renal injury be missed. These patients are the victims of multiple trauma and thus during the evaluation of other intra-abdominal injuries, most of the missed major renal injuries will be detected. When patients who were imaged for associated intra-abdominal injuries are included only 0.03% of significant renal injuries were not identified.[1, 3-5]
3. *Major deceleration injury.* The kidney primarily floats free in a bed of fat contained within the envelope of Gerota's fascia. The kidney is fixed at only two points, the ureter and the vascular pedicle. Because of poor fixation, the kidney can be easily dislocated by sudden acceleration or deceleration. Kidney dislocation can result in tearing of the collecting system at the ureteropelvic junction (UPJ) or tearing of renal artery intima, resulting in partial to complete vessel occlusion. Such injuries can occur with major deceleration, as in head-on motor vehicle accidents (MVA) or falls from great heights, or from marked flexion-extension, as with pedestrian versus motor vehicle. Pediatric patients are particularly prone to this mechanism of injury.[2,6] In general, all rapid deceleration injuries warrant renal imaging even in the absence of hematuria.

4. *Microscopic or gross hematuria after penetrating flank, back or abdominal trauma; or missile path in line with the kidney*.
5. *Pediatric trauma patient with significant microscopic or gross hematuria.* [7] In comparison to adults, a child's kidney is relatively much larger for its body size. The kidneys are also not as well protected, with perirenal fat that is usually scant and lower ribs that are incompletely ossified. Therefore, children are particularly prone to injury. However, the majority of blunt renal injuries are contusions that require no active therapy.[1,3-5,7] Hypotension is often an unreliable predictor of significant renal injury, as children can maintain a normal blood pressure despite extensive blood loss.[6] Traditionally, all children with any degree of microscopic hematuria after blunt trauma have undergone renal imaging. Morey et al[7] in a meta-analysis of all reported series of children with hematuria and suspected renal injury, however, noted that only 2% (11 of 548) of patients with insignificant microscopic hematuria (<50 RBC/HPF) had a significant renal injury. Furthermore, these 11 patients all had other significant injuries that required abdominal and thus renal imaging. They concluded that in children in stable condition with gross or significant microscopic hematuria (>50 RBC/HPF), or with moderate to severe multisystem trauma (regardless of the hematuria degree) the suspicion for significant renal injury arises, and should thus be imaged. Patients who do not initially undergo renal imaging who have persistent or worsening hematuria should also be imaged. Although renal pedicle injuries can occur without hematuria, they are likely to be associated with severe multisystem trauma that requires abdominal imaging anyway. For suspected renal injury, computed tomography is the best study for staging a solid organ injury.[8,9]
6. *Associated injuries suggesting underlying renal injury.* Blunt trauma and a flank ecchymosis, lumbar vertebral or transverse process fractures, lower rib (11th or 12th) fractures and severe mechanism of injury. Another indication for imaging is a penetrating flank or abdominal injury where the entrance and exit sites (or radio-opaque density) are in the path of the kidney, regardless of the degree of hematuria.

### IMAGING STUDIES

In our experience at San Francisco General, 90% of renal injuries result from blunt trauma, while only 10% result from penetrating trauma.[1] Of these renal injuries, only 4% of blunt trauma and 67% of penetrating trauma are significant renal injuries (grade 2 to 5). The majority of renal injuries are grade 1 (renal contusions) that result from blunt trauma. Grade 1 renal injuries heal spontaneously without adverse sequelae and thus require no staging or active treatment.[3-5] In order to make appropriate management decisions, significant renal injuries (namely, grade 2-5) when possible, should undergo complete radiographic staging.[1] Staging of renal injuries permits selective management based on the extent of renal injury and the patient's clinical status.[10]

**Intravenous Urography.** All penetrating renal and hemodynamically unstable blunt renal trauma patients who require immediate surgical exploration should undergo a one-shot high-dose intravenous urogram (IVU) prior to any renal exploration.[1] A one-shot trauma IVU consists of 2ml/kg of body weight of standard 60% ionic or non-ionic contrast injected intravenously, followed by a single abdominal radiograph 10 minutes later. No scout film is necessary. In children, 2-3 ml/kg of non-ionic contrast is preferred.[11] For a satisfactory study, a systolic blood pressure above 90 is needed. In order to save time, the contrast can be injected at the time of the initial resuscitation. Unstable patients who are emergently taken to the OR, should be stabilized first and undergo a one-shot IVU in the OR once they are stabilized.

The major limitation of the IVP is that it can seldom, in itself, define the full extent of the injury.[10]

The purpose of the IVU is to determine the presence of two functioning renal units, the presence and extent of any urinary extravasation, and in penetrating injuries, the likely course of the missile. Radiopaque markers (paper clips) taped to the skin at the bullet entrance and exit sites help predict the likelihood of the kidney in the missile's path. The IVU is highly accurate for establishing the presence or absence of renal injury.[12] When it comes to staging parenchymal injury, however, the IVU is usually nonspecific and not sensitive.[13,14] An abnormal or equivocal IVU warrants further exploration or radiographic staging. For the hemodynamically stable patient, further and more accurate staging can be achieved by a CT scan.[15,16] For the unstable patient with an abnormal IVU, surgical exploration is warranted.

**Computed Tomography.** For the stable patient, renal injury can be most accurately and completely imaged and staged by computed tomography (CT).[15,16] CT imaging is both sensitive and specific for demonstrating parenchymal lacerations, urinary extravasations, delineating segmental parenchymal infarcts, and for determining the size and location of the surrounding retroperitoneal hematoma, and/or associated intra-abdominal injury (spleen, liver, pancreas, and bowel).

CT imaging has largely replaced the once standard IVU and arteriogram. In the acute setting, CT has completely replaced arteriography because it can also accurately delineate segmental and major arterial injuries. The present role of arteriography, however, is with delayed renal bleeding or delayed arteriovenous fistula formation where super selective arterial embolization is utilized.[19] Renal artery occlusion and global renal infarct are noted on CT by lack of parenchymal enhancement or a persistent cortical rim sign. Although reliable for demonstrating renal infarct, the down side to using the rim sign is that it is usually not seen until at least 8 hours after injury.[17]

In recent years, fast scanning and image reconstructing helical CT scanners have been introduced. Turn around times for abdominal trauma imaging is now in the 10 minute range. Seventy to 90 seconds before initiating the helical CT, 150-180 cc of intravenous contrast is given at 2-4 cc per second. Helical CT imaging is so quick (usually under 2 minutes) that only the arterial phase (20-30 seconds) and the early cortical phase (40-70 seconds) of the kidney are obtained. Arterial phase imaging helps delineate any renal artery injury, while the early cortical phase will still miss most parenchymal injuries. Therefore, in order to complete the proper evaluation and staging of renal injuries, later imaging in the nephrogram phase (>80 seconds) are needed to detect renal parenchymal and venous injury, while delayed images (2 to 10 minutes) are often required to detect urine and blood extravasation. On delayed CT images extravasated urine can be distinguished from blood in that it accumulates, while extravasated arterial contrast dilutes out after the bolus of contrast is stopped.[8,11]

**Ultrasonography.** Experience with the evaluation of suspected acute renal traumatic injury by ultrasound has been primarily from Europe.[18] In well-trained and experienced hands, renal lacerations and hematomas can be reliably identified and delineated. Limitations of ultrasound include an inability to distinguish fresh blood from extravasated urine and an inability to identify vascular pedicle injuries or segmental infarcts. Only with close color and pulsed Doppler interrogation can a vascular injury be diagnosed. Furthermore, concomitant rib fractures, bandages, intestinal ileus, open wounds or morbid obesity will severely limit renal visualization. In general, the accuracy of ultrasound for evaluating the retroperitoneum is variable, time consuming and highly operator dependent. We therefore do not advocate the routine use of ultrasound for screening acute renal trauma. Sonography, however, has proven useful and reliable for evaluating blunt intra-abdominal injuries by detecting the presence of hemoperitoneum. In the hemodynamically unstable patient, it is used as a less invasive replacement for the diagnostic peritoneal lavage. In the stable blunt trauma victim, ultrasound is used to direct patients to CT imaging when hemoperitoneum is noted and those with negative findings to observation.

**Arteriography.** With the advent of accurate and quick computed tomography imaging the uses for arteriography with renal trauma have diminished. Renal arteriography does provide the opportunity to stage the injury and, if necessary to embolize bleeding points at the same time.[12] However, in the acute setting, we rarely employ renal arteriography and embolization for renal trauma because it is time-consuming and patients with active bleeding need to undergo immediate exploratory laparotomy. Furthermore, during celiotomy, the kidney can be explored and surgically reconstructed. Arteriography and superselective embolization continue to play an important role in the evaluation and treatment of symptomatic post-traumatic arterio-venous fistulas or persistent delayed renal bleeding.[19]

### INJURY SCALING

In order to determine the appropriate management for a renal injury, the renal injury first needs to be accurately staged. The American Association for the Surgery of Trauma has defined renal trauma into five grades: Grade 1. Renal contusion or nonexpanding subcapsular hematoma without a parenchymal laceration. Grade 2. Nonexpanding perirenal hematoma or a renal cortex laceration (<1cm) without urinary extravasation. Grade 3. Renal cortex laceration (>1cm) and no urinary extravasation. Grade 4. Renal cortical laceration extending into the collecting system (as noted by contrast extravasation), or a segmental renal artery or vein injury (noted by a segmental parenchymal infarct), or main renal artery or vein injury with a contained hematoma Grade 5. Shattered kidney, avulsion of the renal pedicle, or thrombosis of the main renal artery.[20]

### INDICATIONS FOR RENAL EXPLORATION

In order to select a renal injury for nonoperative management, the injury needs to be imaged and accurately staged. An incompletely staged renal injury requires surgical exploration. Not all penetrating renal injuries require surgical exploration. In our experience at San Francisco General Hospital (SFGH), 77% of renal gunshot wounds and 45% of renal stab wounds are explored.[21] Of blunt renal injuries, we explore a conservative 1.9%. The use of the improved imaging technique of CT has largely been responsible for the decreased rate of renal explorations at our institution. Guidelines for the surgical exploration of the injured kidney vary. The only absolute indication for surgical renal exploration is a patient with external trauma and persistent renal bleeding.[10,22]

### Blunt Trauma

Algorithms that we employ for the evaluation and management of blunt renal injuries among adult patients is detailed in Figure 1 and for pediatric patients (< 16 years old) is detailed in Figure 2.

### Adult Blunt Renal Trauma

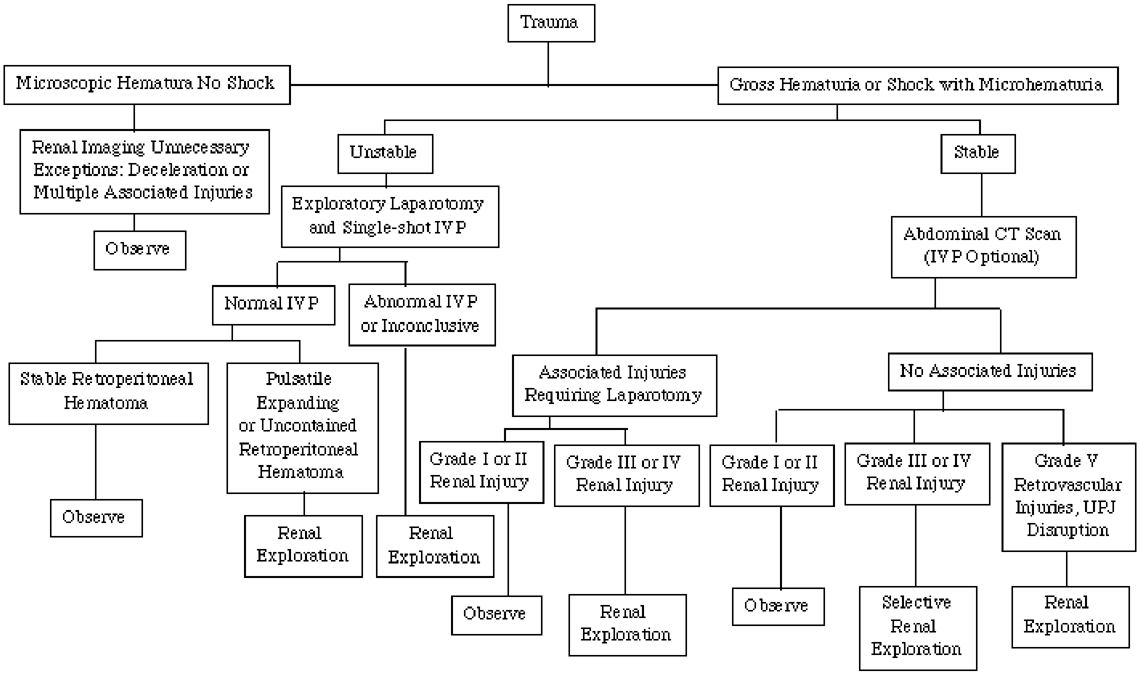

**FIGURE 1**

### Pediatric Blunt Renal Trauma

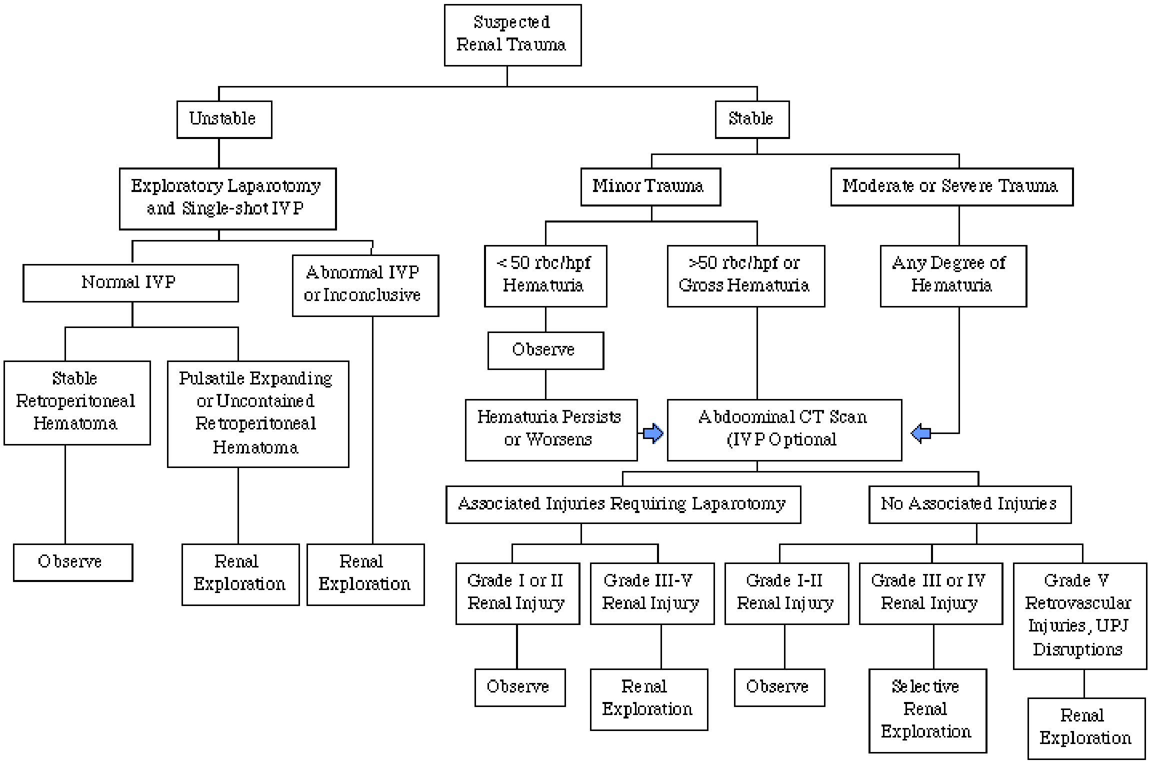

**FIGURE 2**

### Absolute Indications

### *Persistent Renal Bleeding*

Signs of continued renal bleeding are a pulsatile, expanding or uncontained retroperitoneal hematoma.[22] Another sign is avulsion of the main renal artery or vein as noted by CT or arteriography.

### Relative Indications

### *Nonviable Tissue*

Substantial devitalized renal parenchyma (>25%) is a relative indication for exploration. Husmann and Morris[23] have noted that injuries with significant nonviable renal tissue (25-50%) associated with parenchymal laceration that are managed nonoperatively have a high complication rate (82%). Husmann et al[9] further compared the results of the nonoperative and surgical management of major renal lacerations and devitalized renal fragments after blunt trauma. Their findings demonstrated that when such renal injuries are associated with an intraperitoneal organ injury, the post-injury complication rate is much higher unless the kidney is surgically explored and repaired. By surgically repairing such injuries, they reduced the overall morbidity from 85 to 23%. Concomitant pancreatic and bowel injuries were particularly associated with higher rates of infected urinomas and abscesses. Furthermore, since nearly all blunt trauma patients with intraperitoneal organ injuries will undergo celiotomy by the general surgeon, this offers the opportunity to explore and repair the kidney with such major parenchymal injuries.

Patients with a major devitalized segment, associated with urinary extravasation, extensive renal injury, and a large retroperitoneal hematoma, even without intraperitoneal injury, should also be particularly considered for renal exploration.[10] In our experience, when renal exploration is undertaken judiciously and with the goal of preserving renal tissue, 88% of kidneys can be salvaged with a complication rate below 10%.[21]

### Urinary Extravasation

Urinary extravasation in itself does not demand surgical exploration. Extravasation confirms the diagnosis of a major renal injury. Persistent extravasation or signs of sepsis usually require intervention. In general, urinary extravasation will resolve spontaneously in the majority of patients with blunt trauma. In select patients, expectant management does not reduce the renal salvage rate or prolong hospitalization. Nonoperative therapy may also require delayed intervention. However, the usual complications of urinoma and persistent urinary leak can be successfully managed by either percutaneous or endoscopic techniques, and thus avoid celiotomy and renal exploration.[9,24] Matthews et al[25] has reported that patients with major renal injury and urinary extravasation who are managed conservatively, urinary extravasation spontaneously resolved in 87%. Extravasation persisted in 13%, and was successfully managed endoscopically (eg double-J stent). Overall hospitalization lasted 8 days and was not prolonged by the need for delayed intervention.

Ureteropelvic junction (UPJ) injuries will rarely heal spontaneously and thus are often best managed by surgical repair at the time of injury. Conservative management of such injuries are fraught with persistent urine leakage, urinoma formation, ileus and infection.

### Incomplete Staging

Only complete definition of the renal injury by appropriate imaging studies will permit the selection of nonoperative management.[10] Incomplete staging demands either further imaging or renal exploration and reconstruction. In the unstable patient who requires emergent celiotomy, the kidney can only be imaged by a one-shot IVU on the operating room table. The nephrogram of the injured kidney is often poorly opacified due to the injury and worsened by any hemodynamic instability. In so doing, the full extent of the injury is indeterminate. In such circumstances, we explore the kidney after obtaining proximal vascular control. The unexpected finding of a retroperitoneal hematoma upon celiotomy should be evaluated by an on table one-shot IVU. If the IVU results are abnormal or indeterminate or the kidney is persistently bleeding, then the kidney should be explored.

### Arterial Thrombosis

Major deceleration injuries can result in stretching on the renal artery and tearing of the vessel intima, resulting in thrombosis of the main renal artery or its segmental branches and thus causing infarction of the renal parenchyma. Prompt diagnosis and the time till operation of a unilateral complete arterial thrombosis is vital to salvaging the kidney.[26] The rate of renal salvage is remote after 12 hours of ischemia.[27] If the contralateral kidney is normal, there is some controversy as to whether to attempt revascularization or to observe. If renal ischemia exceeds 12 hours, the kidney should be allowed to slowly atrophy. Nephrectomy should be performed here only if delayed celiotomy is being performed for an associated injury or if persistent hypertension develops postoperatively. Bilateral complete renal artery thrombosis or a solitary kidney demands more immediate exploration and revascularization.

### Penetrating Trauma

Again the only absolute indication for exploration is persistent renal bleeding.[28,29] An algorithm for the evaluation and management of penetrating renal injuries is detailed in Figure 3. Nearly all penetrating renal injuries should be managed surgically. The exception are stable patients with no missile penetration of the peritoneum where the injury is well staged by computed tomography. Wessels et al[29] has shown that gunshot wound victims who have no intraabdominal organ injury and a demonstrated grade 1-2 renal injury, when managed conservatively are relatively complication free. In sharp contrast, one out of every four expectantly managed grade 3-4 injuries were complicated by a delayed renal bleed. Stab wounds posterior to the posterior axillary line are less likely to have an associated visceral injury. When the diagnostic peritoneal lavage or CT scan is negative for intraperitoneal organ injury and the renal injury not severe, observation of the renal injury may be appropriate.

Most abdominal penetrating injuries will undergo celiotomy by the general surgeons. Presence of an unexpected retroperitoneal hematoma upon exploratory laparotomy where the renal injury has not been fully staged radiographically usually warrants renal exploration.

### TECHNIQUES OF RENAL EXPLORATION AND REPAIR

We achieve primary vascular control prior to all renal explorations in the manner as initially described by Scott and Selzman[30] and later detailed by McAninch et al,[31] who demonstrated a sharp reduction in the nephrectomy rate by routinely obtaining proximal vascular control. For vascular control, we prefer to isolate the ipsilateral renal artery and vein individually with vessel loops. The kidney is then exposed by incising Gerota's fascia lateral to the colon. When brisk bleeding is encountered, the renal artery is temporarily occluded with Rummel tourniquets. Warm ischemic time should not greatly exceed 30

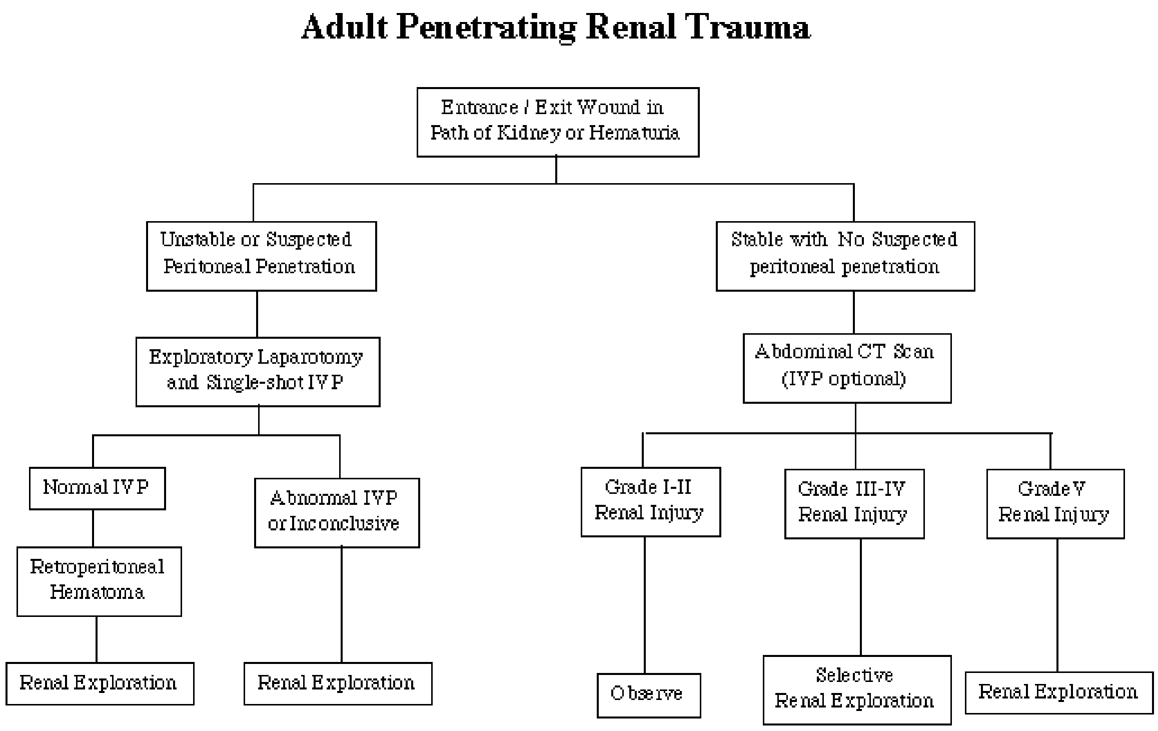

**FIGURE 3**

minutes, in order to avoid permanent renal ischemic damage.[32] If bleeding persists, we then occlude the renal vein by Rummel tourniquet, in order to eliminate back bleeding. Temporary occlusion of the renal artery is needed in patients with renal vascular injuries, those in shock, and those with large or expanding retroperitoneal hematomas.

### Renal Reconstruction

In the absence of persistent hemodynamic instability or coagulopathy, renal reconstruction is safe and effective.[21] The method of kidney reconstruction is dictated by the degree and location of the injury, and not by the associated intraabdominal injuries. In the face of concomitant major pancreatic or colonic injuries with frank fecal contamination, renal reconstruction is successful, with only a slightly increased complication rate.[33,34] The reconstructive principles for renal injures are:[28] 1. Adequate and broad exposure of the kidney and injured area. 2. Temporary vascular occlusion for brisk renal bleeding not well controlled by manual compression of the parenchyma. 3. Sharp excision of all nonviable parenchyma. 4. Meticulous hemostasis (particularly, arterial). 5. Watertight closure of the collecting system. 6. Parenchymal defect closure by approximation of the capsular/parenchymal edges over a Gel-foam bolster or coverage with omentum, perinephric fat, peritoneum or polyglycolic acid mesh. 7. Interposition of a omental pedicle flap between any vascular, colonic, or pancreatic injury and the injured kidney. 8. Ureteral stent placement for a renal pelvis or ureteral injury. 9. Retroperitoneal drain placement. We prefer to use a Penrose drain. Unless drainage is excessive, we remove the Penrose drain after 48 hours. We also always separately drain the urinary tract injury and the pancreatic injury.

### Indications for Nephrectomy

When proximal vascular control is initially achieved before all renal explorations, nephrectomy is required in <12% of cases.[21] When primary vascular control is not achieved and massive bleeding encountered, in the rush to control bleeding, a kidney that could have been salvaged is unnecessarily sacrificed. Overall, nephrectomy is required when the patient is persistently hemodynamically unstable, and thus is a life saving maneuver. Other indications for nephrectomy are grade 5 injuries that are deemed irreparable, such as major vascular pedicle injury, particularly on the right.[35]

### COMPLICATIONS AFTER RENAL TRAUMA

Complications which follow renal trauma are dependent upon the grade of the initial renal injury and the method of management. In most cases, resulting complications are usually of minimal long-term morbidity, can be successfully managed by endourologic and percutaneous techniques, and do not significantly prolong the mean days of hospitalization.[25,36]

Early complications, those that occur within one month of injury are urinoma, delayed bleeding, urinary fistula, abscess, and hypertension. Prolonged urinary extravasation is the most common complication after renal trauma.[1,25] Urinomas occur in <1% of renal trauma cases. Small, uninfected, and stable collections do not require intervention. Larger collections are usually successfully managed by the endoscopic or percutaneous placement of a ureteral/nephrostomy tube. Delayed renal bleeding most commonly occurs within 2 weeks of injury. When bleeding is heavy or symptomatic, transfusions, angiography and superselective embolization[19] may be required. Urinary fistulas can occur in association with an undrained collection or from large segments of devitalized renal parenchyma. Abscesses of the retroperitoneum are associated with ileus, high fever and sepsis. Most collections can be easily drained percutaneously. The extent of the abscess and the presence of loculations are well delineated by CT imaging. Hypertension in the early postoperative period is usually renin-mediated, transient, and does not require any treatment.

Late complications after renal trauma are hydronephrosis, arteriovenous fistula, pyleonephritis, calculus formation and delayed hypertension. Scarring in the region of the renal pelvis and ureter after renal trauma can result in urinary obstruction and subsequently lead to stone formation and chronic infections. Arteriovenous fistula more commonly occur after a renal stab wound and can present with delayed bleeding.[19] Angiography will be able to determine the size and location of the fistula. In most cases, vessel embolization will successfully close the fistula. Long-term hypertension from renal trauma is a rare complication that is overdiagnosed. In our experience at SFGH, sustained hypertension occurred in only 0.2% of cases.[1] The etiology for hypertension after renal injury is renal ischemia which stimulates the renin-angiotensin axis. Long-term follow-up of renal trauma patients is important in order not to miss these late complications which are often of insidious onset and silent progression.

### REFERENCES

1. Miller KS, McAninch JW: Radiographic assessment of renal trauma: Our 15 year experience. J Urol 154:352, 1995.
2. Pollack HM, Wein AJ: Imaging of renal trauma. Radiology 172:297, 1989.
3. Cass AS, Luxenberg M, Gleich P, Smith CS. Clinical indications for radiographic evaluation of blunt renal trauma. J Urol 136:370, 1986.
4. Eastman JA, Wilson TL, Ahlering TE: Radiographic evaluation of adult patients with blunt renal trauma. J Urol 148:266, 1992.
5. Hardeman SW, Husman DA, Chin HKW, Peter PC: Blunt urinary tract trauma: Identifying those patients who require radiological diagnostic studies. J Urol 138:99, 1987.
6. Stein JP, Kaji DM, Eastman J, et al: Blunt renal trauma in the pediatric population: indications for radiographic evaluation. Urology 44:406, 1994.

7. Morey AF, Bruce JE, McAninch JW: Efficacy of radiographic imaging in pediatric blunt renal trauma. J Urol 156:2014, 1996.
8. Shuman WP: CT of blunt abdominal trauma in adults. Radiology 205:297, 1997.
9. Husmann DA, Gilling PJ, Perry MO, et al: Major renal lacerations with a devitalized fragment following blunt abdominal trauma: A comparison between nonoperative (expectant) versus surgical management. J Urol 150:1774, 1993.
10. McAninch JW: Renal trauma. J Urol 150:1778, 1993.
11. Mercader VP, Gatenby RA, Curtis BR: Radiographic assessment of genitourinary trauma. Trauma Q 13:129, 1996.
12. Eastman JA, Wilson TG, Ahlering TE: Urological evaluation and management of renal proximity stab wounds. J Urol 150:1771, 1993.
13. Bergren CT, Chan FN, Bodzin JH: Intravenous pyelogram results in association with renal pathology and therapy in trauma patients. J Trauma 27: 515, 1987.
14. Carroll PR, McAninch JW: Staging of renal trauma. Uro Clin N Am 16:193-201, 1989.
15. McAninch JW and Federle, MP: Evaluation or renal injuries with computed tomography. J Urol, 128:456, 1982.
16. Bretan PN, Jr., McAninch JW, Federle MP, Jeffrey RB, Jr.: Computerized tomographic staging of renal trauma: 85 consecutive cases. J Urol 136:561, 1986.
17. Kamel IR, Berkowitz JF: Assessment of the cortical rim sign in post-traumatic renal infarction. J Comp Assis Tomogr 20:803, 1996.
18. Furschegger, Egender G, Jaske G. The value of sonography in the diagnosis and follow-up of patients with blunt renal trauma. R J Urol 62:110, 1988.
19. Heyns CF, Van Vollenhoven P: Increasing role of angiography and segmental artery embolization in the management of renal stab wounds. J Urol 147:1231,1992.
20. Moore EE, Shackford SR, Pachter HL et al: Organ injury scaling: Spleen, liver, and kidney. J Trauma 29:1664, 1989.
21. McAninch JW, Carroll PR, Klosterman PW, et al: Renal reconstruction after injury. J Urol 145:932, 1991.
22. Holcroft JW, Trunkey DD, Minagi H, et al: Renal trauma and retroperitoneal hematomas-indications for exploration. J Trauma 15:1045, 1975.
23. Husman DA, Morris JS: Attempted nonoperative management of blunt renal lacerations extending through the cortico-medullary junction; The short term and long term sequelae. J Urol 143:682, 1990.
24. Cheng DL, Lazan D, Stone N: Conservative treatment of type III renal trauma. J Trauma 36:491, 1994.
25. Mathews LA, Smith EM, Spirnak JP: Nonoperative treatment of major blunt renal lacerations with urinary extravasation. J Urol 157:2956, 1997.
26. Carroll PR, McAninch JW, Klosterman P, Greenblatt M: Renovascular trauma: Risk assessment, surgical management, and outcome. J Trauma 30: 547, 1990
27. Cass AS, Luxenberg M: Conservative or immediate surgical management of blunt renal injuries. J Urol 130:11, 1983
28. McAninch JW, Carroll PR, Armenakas NA, Lee P: Renal gunshot wounds: Methods of salvage and reconstruction. J Trauma 35: 279, 1993.
29. Wessells H, McAninch JW, Meyer A, Bruce JE: Criteria for nonoperative treatment of significant penetrating renal lacerations. J Urol 157:24, 1996.
30. Scott RF, Selzman HM: Complications of nephrectomy: Review of 450 patients and a description of a modification of the transperitoneal approach. J Urol 95:307, 1966.
31. McAninch JW, Carroll Pr: Renal trauma: Kidney preservation through improved vascular control, a refined approach. J Trauma 22:285, 1982.
32. Carroll PR, Klosterman P, McAninch JW: Early vascular control for renal trauma: A critical review. J Urology 141:826, 1989.
33. Rosen MA, McAninch JW: Management of combined renal and pancreatic trauma. J Urol 152, 22, 1994.
34. Wessells H, McAninch JW: Effect of colon injury on the management of simultaneous renal trauma. J Urol 155:1852, 1996.
35. Nash PA, Bruce JE, McAninch JW: Nephrectomy for traumatic renal injuries J Urol 153:609,1995.
36. Roberts AR, Belitsky P, Lannon SG, et al: Conservative management of renal lacerations in blunt trauma. Can J Surg 30:253, 1987.

---

**This article should be referenced as follows:**

Brandes, S.B. and McAninch, J.W. (2004) Renal trauma: a practical guide to evaluation and management. *TheScientificWorldJOURNAL* **4 (S1)**, 31–40.

**Handling Editor:**

Anthony Atala, Principle Editor for *Urology* — a domain of *TheScientificWorldJOURNAL*.

---
